# Molecularly tunable thin-film nanocomposite membranes with enhanced molecular sieving for organic solvent forward osmosis

**DOI:** 10.1038/s41467-020-15070-w

**Published:** 2020-03-05

**Authors:** Bofan Li, Susilo Japip, Tai-Shung Chung

**Affiliations:** Department of Chemical & Biomolecular Engineering, National University of Singapore, 4 Engineering Drive 4, Singapore, 117585 Singapore

**Keywords:** Organic molecules in materials science, Polymers, Polymers

## Abstract

Thin-film nanocomposites (TFN) functionalized with tunable molecular-sieving nanomaterials have been employed to tailor membranes, with an enhanced permeability and selectivity. Herein, water-soluble hollow cup-like macrocyclic molecules, sulfothiacalix[4]arene (STCAss) and sulfocalix[4]arene (SCA), are ionically bonded into the polyamide network to engineer the molecular-sieving properties of TFN membranes for organic solvent forward osmosis (OSFO). Introducing both STCAss and SCA into the polyamide network not only increases the free volume, but also reduces the thickness of the TFN layers. Combining with their molecularly tunable size of the lower cavities, both STCAss and SCA enable the TFN membranes to size exclusively reject the draw solutes, but only STCAss-functionalized membrane has an ethanol flux doubling the pristine one under the FO and PRO modes in OSFO processes; leading the functionalized polyamide network with remarkable improvements in OSFO performance. This study may provide insights to molecularly functionalize TFN membranes using multifunctional nano-fillers for sustainable separations.

## Abstract (web summary)

Thin-film nanocomposites (TFN) nanomaterials have been employed to tailor permeability and selectivity in membranes, but achieving effective separation at large flux retains challenging. Here, the authors use calix[4]arene derivatives which are ionically bonded to a polyamide network to engineer the molecular-sieving properties of TFN membranes for organic solvent forward osmosis (OSFO).

## Introduction

Organic solvents are intensively employed in pharmaceutical syntheses. A green and efficient practice to recycle waste organic solvents instead of incineration is imperative for earth sustainability^1^. However, traditional separation processes, such as distillation and evaporation, suffer from high energy consumption, large footprints, and environmental unfriendliness. Membrane-based separation to recycle waste organic solvents is emerging in recent decades by means of hydraulic or osmotic pressure as the driving force^2–8^. Among them, organic solvent nanofiltration (OSN) is widely studied but the high operating pressure and fouling tendency may increase the capital and operating costs^7^. Recently, a novel process, organic solvent forward osmosis (OSFO), which was initially proposed by Lively and Sholl^8^, and then demonstrated by Cui and Chung^9^, is promising to lower the fouling tendency and treat the highly concentrated feed solutions. Similar to forward osmosis (FO) in water reuse processes^10^, both valuable products and organic solvents can be recovered from pharmaceutical streams. A membrane with both high permeability and selectivity is desirable to yield good OSFO performance. Nevertheless, little work has been done in the field of OSFO, especially for the development of OSFO membranes.

Hydration Technology Innovations (HTI) was the pioneer in developing FO membranes from cellulose acetate (CA), where the selective and support layers were formed integrally via phase inversion^11,12^. Afterward, thin-film composite (TFC) membranes for seawater reverse osmosis (RO) were modified and adopted for the fabrication of FO membranes^13–16^. The TFC configuration was superior to the asymmetric, integrally skinned, one in HTI CA membranes because its selective layer and porous substrate could be fabricated separately. As a result, TFC FO membranes generally have a higher flux and a lower internal concentration polarization (ICP) than HTI CA membranes. Thin-film nanocomposite (TFN) FO membranes were further developed to improve the performance. Nano-fillers, such as carbon nanotubes^16^, graphene oxides^17^, metal oxide nanoparticles^18,19^, and metal–organic framework^20^, have been incorporated into the polyamide selective layers to provide extra free volumes for solvent transport. However, the weak affinity between nano-fillers and the polyamide network may lead to particle aggregation and sacrifice the selectivity^21^. In addition, the construction of transporting channels by nano-fillers usually compromise the selectivity because of their interference to form a dense polyamide network.

**Fig. 1:**
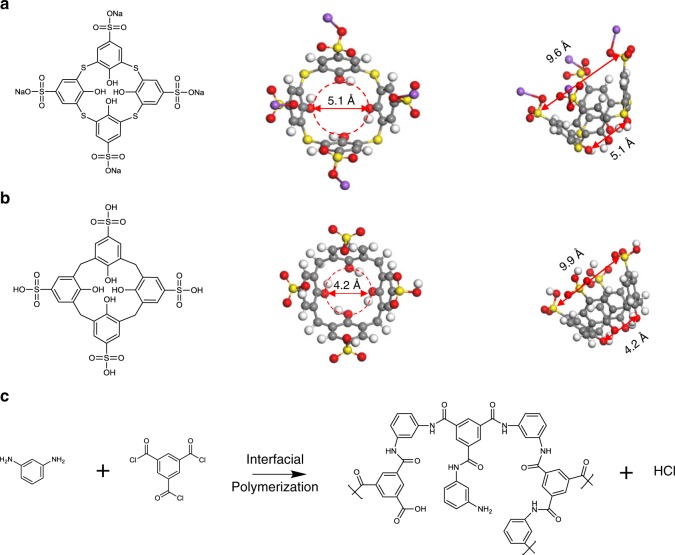
Molecular structures and reaction scheme. Molecular structures of **a** STCAss, **b** SCA, and **c** interfacial polymerization between MPD (aqueous phase) and TMC (oil phase).

Calix[n]arene is a type of close-loop macrocyclic molecules, containing several repeating units of phenolic blocks with a hollow cup-like structure. As illustrated in Fig. 1a, b, the wide upper-rim, narrow lower-rim, and the number of repeating units (*n*) can be molecularly tuned to synthesize various kinds of calix[n]arene^22,23^. Up to now, the exploration of calix[n]arene applications are only limited to a few areas, such as molecular recognition, sensor, catalyst, and gas separation membranes^22–25^.

Inspired by their molecular-tunability and molecular-sieving characteristics, we aim to design TFN membranes by ionically-bonding sulfothiacalix[4]arene (STCAss) and sulfocalix[4]arene (SCA) in the polyamide network, and examine their potential for OSFO. Thiacalix[n]arene is a subgroup of calix[n]arene where the methylene bridge between each phenolic block is replaced by sulfur atoms, which may alter the lower-rim dimension and ultimately the size-sieving properties. Since both STCAss and SCA have multifunctionalities, they may interact with the polyamide layer closely and affect its formation and separation performance. Therefore, the objectives of this work are to (1) functionalize the polyamide network by incorporating close-loop size-selective macrocyclic molecules, and (2) simultaneously improve the permeability and selectivity of the TFN membranes for OSFO processes.

## Results

### Surface morphologies

**Fig. 2:**
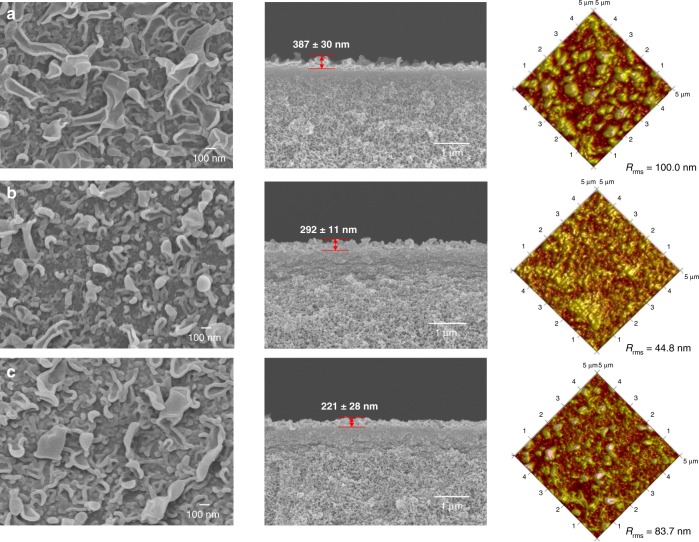
Surface morphologies. FESEM and AFM images of the polyamide layers for **a** TFC-0, **b** TFN-STCAss-1.5, and **c** TFN-SCA-1.5.

Figure 2 presents the surface morphologies of the pristine TFC and TFN membranes. The pristine polyamide layer has a typical ridge-and-valley morphology with relatively big “leaves”. After incorporating STCAss and SCA, the resultant polyamide layers still possess ridge-and-valley morphologies but the “leaves” become smaller. In addition, the thickness of the polyamide layer decreases with the addition of nano-fillers possibly due to the acidic nature of STCAss and SCA, which possess four sulfonate and sulfonic acid groups in one molecule, respectively. Among them, TFN-SCA-1.5 has the thinnest selective layer. The pH values of the m-phenylenediamine (MPD) solutions confirm our hypothesis. The MPD solutions containing 1.5 wt% STCAss and SCA have pH values of ~6.6 and 5.5, respectively, while the pristine MPD solution has a pH value of ~9. Since the sulfonate and sulfonic acid groups in STCAss and SCA may interact with MPD, their presence interferes the interfacial polymerization process^26^. Moreover, the proton dissociated from the sulfonic acid groups would inhibit the HCl generation during the interfacial polymerization because it shifts the reaction equilibrium backward. Both factors result in a lower degree of cross-linking reaction between MPD and trimesoyl chloride (TMC) during the interfacial polymerization, leading to a less crumpled polyamide layer.

A comparison of the surface morphology between TFN membranes embedded with STCAss and SCA indicates that TFN-STCAss-1.5 has smaller “leaves” than TFN-SCA-1.5, although the latter has a slightly lower pH than the former. This may be caused by the Na^+^ ions released from STCAss. The interfacial polymerization reaction generally takes place in two steps: (i) MPD in the aqueous phase diffuses toward the interface and reacts with TMC to form a nascent polyamide layer without the ridge-and-valley structure; (ii) Marangoni convection further prompts the migration of MPD vigorously to react with TMC, thus pushing and bending the nascent polyamide layer and forming a ridge-and-valley structure^27,28^. Since Marangoni convection is affected by the surface tension near the water/hexane interface and the addition of inorganic electrolytes, such as Na^+^ and K^+^, elevates the surface tension of water^29^, the presence of Na^+^ from STCAss in the MPD solution may increase the surface tension near the water/hexane interface, and reduce the Marangoni convection, leading to a smaller “leaves”.

The surface topology of these polyamide layers was further probed by atomic force microscopy (AFM) to determine the surface roughness. As shown in Fig. 2, the order of surface roughness is consistent with the observation from field emission scanning electron microscopy (FESEM) images, which follows TFC-0 > TFN-SCA-1.5 > TFN-STCAss-1.5. This order further confirms the effects of pH and Na^+^ on the formation of polyamide layer during the interfacial polymerization.

### Surface chemistries

**Fig. 3:**
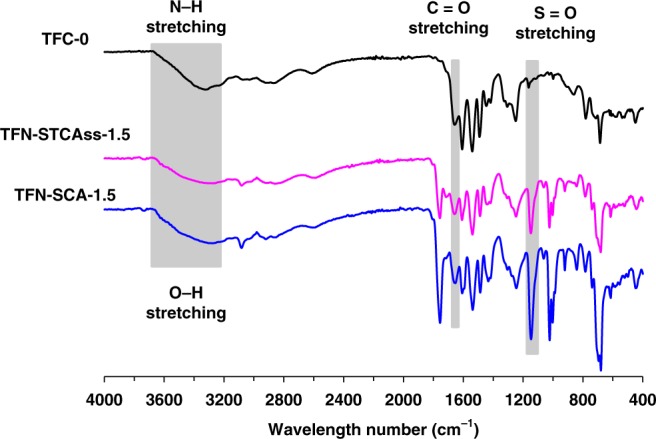
Surface chemistry. ATR-FTIR spectra for TFC-0, TFN-STCAss-1.5, and TFN-SCA-1.5.

The surface chemistries of the pristine and modified polyamide layers were characterized by Fourier transform infrared spectroscopy (FTIR) under the attenuated total reflectance (ATR) mode and X-ray photoelectron spectroscopy (XPS) using free-standing polyamide films. Figure 3 displays the ATR-FTIR spectra of TFC-0, TFN-STCAss-1.5, and TFN-SCA-1.5. The pristine polyamide layer consists of the typical N-H and C=O bonds of amide groups, which show sharp stretching peaks at 3300 cm^−1^ and 1620 cm^−^^1^, respectively. In comparison, after STCAss and SCA incorporation, the sharp N-H stretching peak of the amide group is covered by a broad O-H stretching peak in the range of 3200–3650 cm^−1^, while an S=O stretching peak at 1149 cm^−1^ appears. The O-H and S=O bonds belong to the hydroxyl and sulfonate/sulfonic acid groups of STCAss and SCA, respectively. This indicates the successful incorporation of both STCAss and SCA into the polyamide layers. However, it is difficult to distinguish the difference between TFN-STCAss-1.5 and TFN-SCA-1.5 from the ATR-FTIR spectra because the C-S bond shows a weak peak in FTIR that is usually covered by other peaks. Therefore, XPS is employed to further investigate the surface composition and bonding information of the free-standing TFC and TFN membranes.

Supplementary Table 1 tabulates the surface compositions of these polyamide layers. The S atom can hardly be detected in the pristine polyamide layer although there is a tiny amount of sodium dodecyl sulfate (SDS; 0.2 wt%) in the MPD aqueous solution. By incorporating STCAss and SCA, the S contents in the polyamide layers increase to 0.34 At% and 0.26 At%, respectively. The S content of TFN-STCAss-1.5 is higher than that of TFN-SCA-1.5 because each STCAss molecule consists of 8 S atoms and each SCA molecule possesses only 4 S atoms. The C contents are similar for the three polyamide layers; however, the O contents increase while the N contents decrease after the incorporation of STCAss and SCA. This is due to the fact that STCAss and SCA possess high concentrations of O elements, owing to both hydroxyl and sulfonate/sulfonic acid groups but no N elements.

**Fig. 4:**
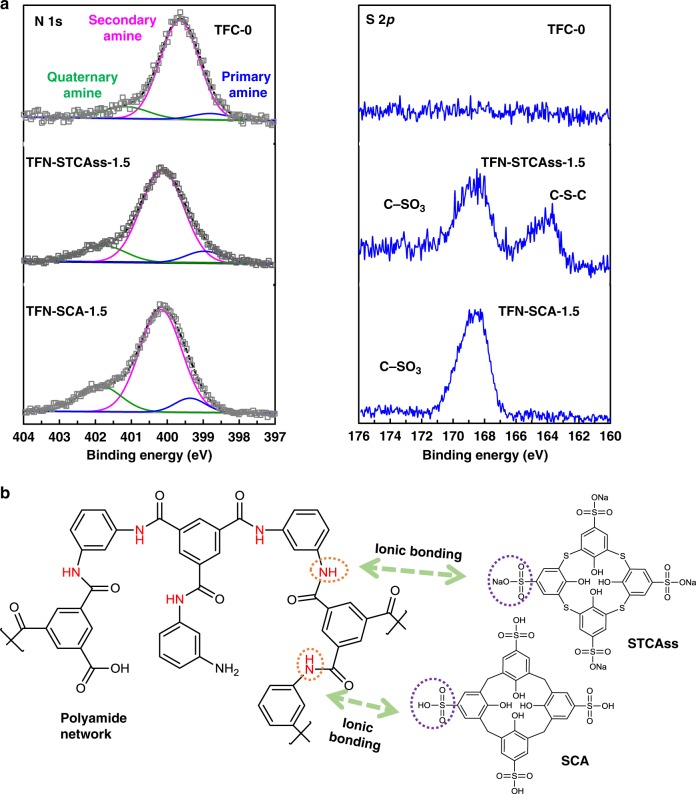
Ionic interactions between the polyamide network and STCAss/SCA. **a** Deconvolution of N 1 *s* and S 2 *p* XPS spectra for TFC-0, TFN-STCAss-1.5, and TFN-SCA-1.5, and **b** illustrations on the interactions between polyamide network and STCAss/SCA.

To further analyze the surface chemistry, the N 1 *s* and S 2 *p* XPS spectra are deconvoluted and displayed in Fig. 4a. For the N peak, there are three fitted peaks; namely, (1) primary amine from the partially cross-linked MPD monomer, (2) secondary amine for the polyamide group, and (3) quaternary amine. The percentages of their peak areas are calculated and listed in Supplementary Table 2. The percentage of the secondary amine decreases significantly with the incorporation of STCAss and SCA, while the contents of both primary and quaternary amines increase slightly. The decrease in secondary amine and the increase in primary amine further confirm the lower cross-linking degrees in these two modified polyamide layers as observed in FESEM. Moreover, the increase in quaternary amine content may be due to the formation of ionic bonding between the sulfonate/sulfonic acid groups and unreacted amine/amide groups. As elucidated in Fig. 1a, b, the sulfonate and sulfonic acid groups in STCAss and SCA are strong electron withdrawing groups; meanwhile, the amine and amide groups tend to donate electrons to them so that ionic bonds are formed, as depicted in Fig. 4b. This ionic bonding not only ensures strong interactions between the incorporated nano-fillers and the polyamide network, but also facilitates the dispersion of STCAss and SCA nano-fillers inside the polyamide layer. As a result, the scanning electron microscopy-energy dispersive X-ray spectroscopy (SEM-EDX) image of S elements in TFN-STCAss-1.5 (Supplementary Fig. 1) shows an even distribution across the membrane surface, indicating a homogenous dispersion of STCAss in the polyamide network. The ionic bonding between the polyamide network and STCAss/SCA would make the developed TFN membranes superior to those conventional TFN membranes in terms of stability and homogeneity because the latter tends to only have physical interactions between the polyamide layer and nano-fillers.

For the S 2 *p* XPS results, there is no obvious peak for the pristine polyamide layer, which is consistent with the surface composition analysis. In contrast, the polyamide layers embedded with STCAss and SCA show two separate peaks and one sharp peak, respectively. The two peaks for TFN-STCAss-1.5 are attributed to C-SO_3_ (168.5 eV) and C-S-C (164 eV), since there are two types of sulfur atoms in STCAss^30^. For TFN-SCA-1.5, since SCA only contains C-SO_3_, there is only one sharp peak observed at 168.5 eV. Thus, by using XPS, TFN-STCAss-1.5, and TFN-SCA-1.5 can be simply distinguished from their S 2 *p* spectra.

### Membrane microstructures

**Fig. 5:**
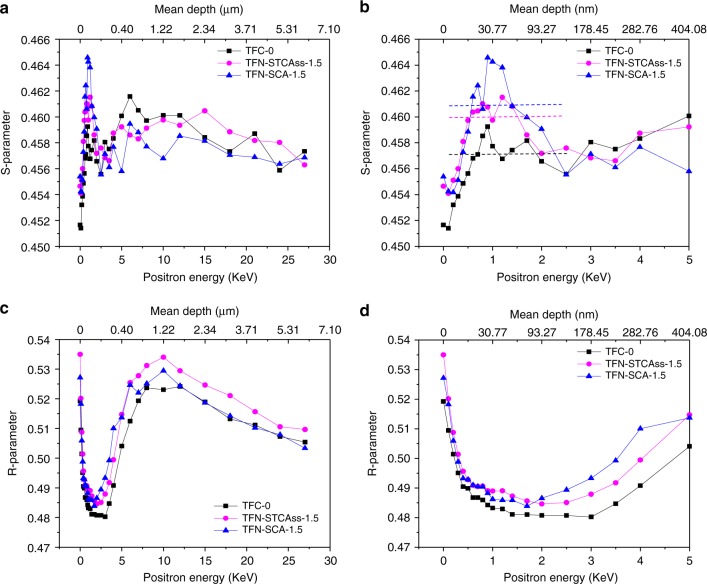
Membrane microstructures. **a**, **b** S-parameters and **c**, **d** R-parameters of TFC-0, TFN-STCAss-1.5, and TFN-SCA-1.5. The dotted lines in **b** indicate the average values of S-parameters of polyamide networks for guiding the view.

In order to investigate the depth profile of microstructures and the evolution of free volume and thickness in polyamide layers, TFC-0, TFN-STCAss-1.5, and TFN-SCA-1.5 were examined by positron annihilation spectroscopy (PAS). As illustrated in Fig. 5, the S- and R- parameters are used to characterize the changes of free volume and micro-voids as a function of positron penetration depth (i.e., membrane thickness), respectively. The S-parameter represents the intensity of free volume, where a larger S-parameter stands for increased free volume cavities and/or a higher free volume. Similarly, the R-parameter describes the pore size distribution, where a larger R-parameter means that the voids (in nm to µm sizes) become larger and/or their quantities increase.

As shown in Fig. 5a, b (enlarged one), the S-parameters of all membranes increase sharply first, which is caused by the back diffusion and scattering of positroniums near the membrane surface^31^. Then, TFC-0 exhibits a different trend from the other two. Its S-parameter fluctuates at a certain value and then increases further. The initial fluctuation represents the dense polyamide layer and the later increase indicates the transition from the dense polyamide layer to the cross-linked polyimide substrate. As the free volume intensity inside the membrane is not ideally homogenous along the membrane depth, the S-parameter normally fluctuates around a certain level instead of a smooth line. In contrast, the S-parameters of TFN-STCAss-1.5 and TFN-SCA-1.5 reach the maximum values first at ~1–1.2 KeV and then decrease at higher incident energy, matching the S-parameter of the substrate in TFC-0 at ~2.5 KeV. Thus, the S-parameter corresponding to the positron energy of 0.5–2.5 KeV represents the polyamide layer. To facilitate easy comparison, eye-guiding lines that represent the average values of S-parameters in the range of 0.5–2.5 KeV, have been added as dotted lines in Fig. 5b. The average values of S-parameters, corresponding to the polyamide layers, follow the order of TFC-0 < TFN-STCAss-1.5 ≈ TFN-SCA-1.5. In addition, there is hardly any overlap between TFC-0 and TFN-STCAss-1.5/TFN-SCA-1.5 in the range of 0.5–2.5 KeV, so the changes in free volume intensity by incorporating STCAss and SCA are considered to be significant. This confirms that the incorporation of STCAss and SCA into the polyamide network can significantly increase its free volume intensity.

The R-parameters of all membranes present a similar trend, as plotted in Fig. 5c, d. The R-parameter decreases at the beginning and reaches the lowest value, followed by a drastic increase, which forms a “V-shape” curve. The initial decrease and subsequent increase imply the existence of a thin dense-selective layer. Thus, the distance between the initial point and the bottom of the “V-shape” can be interpreted as the dense-layer thickness, and the minimum value of the “V-shape” indicates the lowest intensity of voids. Consistent with the observation from FESEM images, the dense-layer thickness follows a descending order of TFC-0 > TFN-STCAss-1.5 > TFN-SCA-1.5. In addition, the pristine TFC membrane has the smallest intensity of voids, while the other two TFN membranes possess a similar intensity of voids. In summary, the pristine polyamide layer has the lowest intensity of free volume and voids as well as the largest thickness; the STCAss-incorporated polyamide layer is slightly thinner and has a relatively larger intensity of free volume and voids; while the SCA-modified polyamide layer possesses similar intensity of free volume and voids as the STCAss-incorporated polyamide layer, but has the thinnest polyamide layer among them.

### OSFO performance and transport properties

**Fig. 6:**
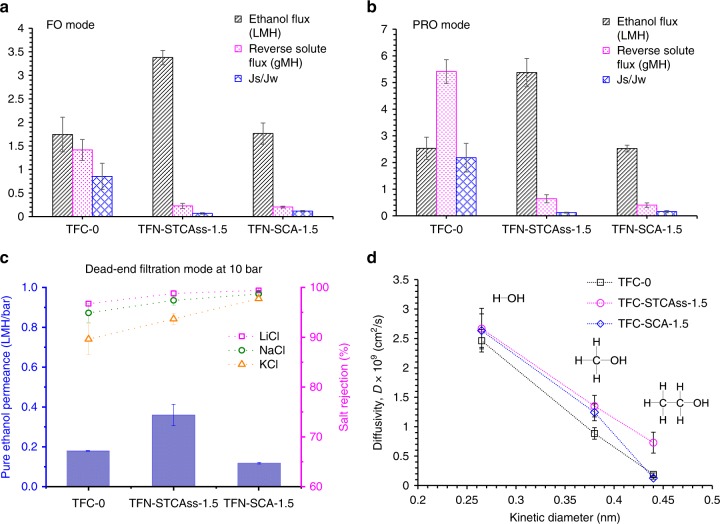
Separation performance and transport properties of TFC-0, TFN-STCAss-1.5, and TFN-SCA-1.5. OSFO performance for **a** FO mode and **b** PRO mode, as well as **c** transport properties and **d** diffusivities of water, methanol, and ethanol in TFC-0, TFN-STCAss-1.5, and TFN-SCA-1.5.

The OSFO performances of TFC-0, TFN-STCAss-1.5, and TFN-SCA-1.5 were quantified under FO and pressure-retarded osmosis (PRO) modes, using pure ethanol and 2 M LiCl in ethanol as feed and draw solutions, respectively. Figure 6a, b shows their ethanol flux (*J*_w_), reverse solute flux (*J*_s_), as well as *J*_s_/*J*_w_. The pristine TFC membrane has a relatively low ethanol flux and a high reverse salt flux under FO and PRO modes, which are not desirable for an FO membrane. In contrast, the STCAss-incorporated membrane has a dramatic increase in ethanol flux under both FO and PRO modes due to its thinner polyamide layer and a larger free volume, as validated by PAS. Surprisingly, TFN-SCA-1.5 has almost the same ethanol flux as TFC-0, although the former has a higher free volume and a thinner polyamide layer than the latter, as revealed by PAS. Meanwhile, the reverse solute flux follows the order of TFC-0 > TFN-STCAss-1.5 > TFN-SCA-1.5. In other words, the STCAss and SCA-incorporated membranes have much lower reverse solute fluxes than the pristine one, which also does not follow the trend of their free volumes observed by PAS.

These interesting phenomena may arise from the fact that the close-loop macrocyclic molecules, i.e., STCAss and SCA, not only function as pore formers but also act as precise molecular sieves that only allow small molecules (i.e., smaller than their cavity sizes) to pass through and reject or block the big ones. According to the molecular structures of STCAss and SCA drawn and optimized by Material Studio^®^, Fig. 1 reveals that the diameters of the lower cavities in STCAss and SCA are ~5.1 Å and 4.2 Å, respectively. Since the kinetic diameter of an ethanol molecule is ~4.4 Å (refs. ^32,33^), ethanol can easily pass through the small opening of STCAss but may not go through SCA. As a consequence, even though TFN-SCA-1.5 has a higher free volume and a thinner polyamide, it exhibits a similar ethanol flux to the pristine TFC membrane. Similarly, the small cavities of both STCAss and SCA may reject Li^+^ and Cl^−^ ions in ethanol although their solvated diameters in ethanol are not known. Nevertheless, the hydrated diameters of Li^+^ and Cl^−^ are 7.64 Å and 6.64 Å, respectively^34^, which are much larger than the small cavity sizes of STCAss and SCA. Hence, the STCAss- and SCA-incorporated membranes reject Li^+^, Cl^−^, and LiCl, and show low reverse solute fluxes in OSFO tests.

In order to confirm our hypothesis, the transport properties of these membranes were investigated by measuring their pure ethanol permeances (*A*) and salt rejections (*R*_s_) under the dead-end filtration mode. Figure 6c shows that TFN-STCAss-1.5 possesses the highest pure ethanol permeance, while TFN-SCA-1.5 has the lowest pure ethanol permeance. The rejection toward LiCl, NaCl, and KCl follows the order of TFN-SCA-1.5 > TFN-STCAss-1.5 > TFC-0, which reconfirms our hypothesis that both small cavities of STCAss and SCA can effectively reject Li^+^, Cl^−^, and LiCl. For all three membranes, the rejections follow the order of LiCl > NaCl > KCl, due to the different sizes of solvated ions in ethanol. In addition, the transport properties of ethanol measured under the dead-end filtration mode are consistent with those under OSFO modes. TFN-STCAss-1.5 always has the highest ethanol flux among these membranes. Although TFC-0 has a slightly higher pure ethanol permeance than TFN-SCA-1.5 under the dead-end filtration mode, their ethanol fluxes under OSFO modes are similar. This may be due to the fact that TFC-0 has a higher reverse solute flux. Thus, the driving force across this membrane is reduced that results in a lower ethanol flux under OSFO tests^35,36^.

The solvent transport mechanism for the developed membranes was also investigated by measuring pure solvent permeances of water, methanol, ethanol, and then calculating their diffusivities in the membranes according to the solution–diffusion model. As shown in Fig. 6d, the solvent diffusivities of all membranes decrease as the solvent molecules become larger. This is in accordance to the diffusivity correlating equations, i.e., diffusivity is inversely related to molecular size. However, the trend of TFN-SCA-1.5 is slightly different from those of TFC-0 and TFN-STCAss-1.5. The former has a much lower ethanol diffusivity than the latter because it has a stronger size-sieving effect to block ethanol, as explained previously.

The tunable molecular-sieving properties of the fabricated nanocomposites were also observed in aqueous solutions, when ethylene glycol (EG), diethylene glycol (DEG), and glucose were selected as probing solutes for aqueous filtration tests (Supplementary Fig. 2). Interestingly, the rejection of TFN-SCA-1.5 toward EG is much larger than those of TFC-0 and TFN-STCAss-1.5. However, the rejections of both TFN-SCA-1.5 and TFN-STCAss-1.5 toward DEG are similar and are only slightly higher than that of TFC-0. Since the diameter of EG is 4.7 Å (ref. ^37^), it may only be excluded by SCA but not by STCAss. However, the diameter of DEG is 5.82 Å, which could be rejected by both SCA and STCAss. Meanwhile, a similar trend in rejections can also be observed for glucose, which is attributed to its diameter of >7 Å. This phenomenon further implies that both STCAss and SCA can molecularly tune the molecular-sieving properties of the fabricated TFNs not only in ethanol but also in aqueous solutions.

### Optimization of STCAss loading

Based on the above results, STCAss is selected as the suitable nano-filler for further studies because it can increase free volume of the polyamide layer, reduce its thickness, and maintain high solute rejection. Thus, the STCAss loading in the MPD solution was varied from 0 to 2.0 wt%, in order to investigate the optimal loading and obtain the best OSFO performance. Supplementary Tables 3 and 4, and Fig. 3 summarize AFM, XPS, and FESEM results, respectively. Generally, an increase in STCAss loading in the MPD solution results in the polyamide surface with a higher S content and a smoother polyamide layer due to the aforementioned acidic effect. The smooth polyamide layer may be desirable to minimize fouling and maintain a sustainable ethanol flux^38^.

**Fig. 7:**
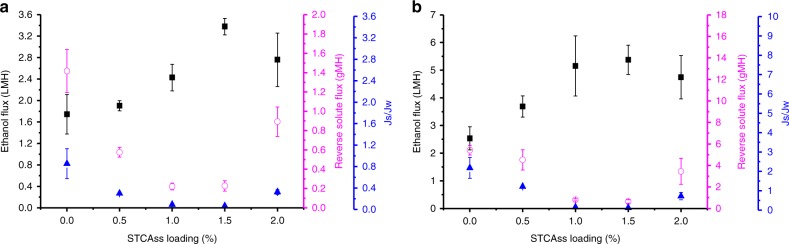
Optimization of STCAss loading. Ethanol flux (closed square), reverse solute flux (open circle), and *J*_s_/*J*_w_ (closed triangle) with increasing STCAss loading for **a** FO mode and **b** PRO mode. The feed solution is pure ethanol and the draw solution is 2 M LiCl in ethanol.

Figure 7 illustrates the OSFO performance under FO and PRO modes as a function of STCAss loading. In both modes, the ethanol flux rapidly increases while the reverse solute flux significantly decreases, with an increase in STCAss loading from 0 wt% to 1.5 wt%, leading to a desirable *J*_s_/*J*_w_ value. The high ethanol flux guarantees a high ethanol recovery while the low reverse solute flux helps in maintaining the driving force across the membrane and ensuring a stable ethanol flux. In addition, the low *J*_s_/*J*_w_ value indicates a low leakage of the draw solute to the feed stream and minimizes the potential hazard caused by the reverse flux of draw solutes. However, the ethanol flux drops and the reverse solute flux bounces back significantly, when an excess amount of STCAss (2 wt%) is embedded in the polyamide layer. This is due to defect formation as evidenced by the increase in surface roughness and the appearance of bulges (Supplementary Table 3, Fig. 3). The TFN formed by incorporating 1.5 wt% STCAss in the MPD solution results in the best OSFO performance. Comparing to the pristine TFC membrane, its ethanol fluxes are almost doubled under both FO and PRO mode. Interestingly, the percentage of increment under the FO mode (93.7%) is slightly lower than that of the PRO mode (112.2%) because of the ICP effect^39^. The former has a lower *J*_s_/*J*_w_ value of 0.07 than the latter of 0.12 using 2 M LiCl as the draw solution, respectively.

## Discussion

We have successfully functionalized the polyamide network with two calix[n]arene, STCAss and SCA for OSFO by incorporating them into the MPD monomer solution via interfacial polymerization. Under FO and PRO modes, the optimized TFN membrane containing 1.5 wt% STCAss in the MPD solution exhibits higher ethanol fluxes (FO: 3.38 L m^−2^ h^−1^ (LMH) and PRO: 5.37 LMH) and lower reverse solute fluxes (FO: 0.23 g m^−2^ h^−1^ (gMH) and PRO: 0.64 gMH) than the pristine TFC membrane (FO: 1.74 LMH, 1.42 gMH and PRO: 2.53 LMH, 5.41 gMH). FESEM, AFM, and PAS results confirm the synergetic effects of STCAss on the polyamide network due to: (i) the thinner and smoother polyamide layer caused by the acidic nature of STCAss, (ii) the ionic bonding formed between the sulfonate groups of STCAss and the amide/amine groups of the polyamide network, (iii) the increased free volume in the polyamide network, and (iv) the size-exclusion effect from the hollow cup-like structure of STCAss.

As the TFN-STCAss-1.5 exhibits the optimal OSFO performance, its applicability to simultaneously concentrate pharmaceuticals and reclaim organic solvents is further tested using two feed solutions of 2 g L^−1^ paracetamol (MW = 151.2 g mole^−1^) and tetracycline (MW = 444.4 g mole^−1^) in ethanol and 2 M LiCl as the draw solutions. The membrane exhibits stable ethanol fluxes of 3.41 LMH and 3.26 LMH, with rejections of 96.1% and 99.6% toward paracetamol and tetracycline, respectively. To compare with OSN or low-pressure organic solvent reverse osmosis (OSRO), filtration tests were conducted using 0.2 g L^−1^ paracetamol and tetracycline solutions as feed solutions for TFN-STCAss-1.5. As shown in Supplementary Table 5, the results clearly suggest that the incorporation of STCAss into the polyamide layer has great potential to molecularly design TFN membranes for OSFO, OSN, and low-pressure OSRO, in order to simultaneously concentrate pharmaceutical products and recover organic solvents.

This study may provide a concept to molecularly design TFN membranes with the aid of macrocyclic molecules, such as STCAss to simultaneously improve permeability and selectivity for OSFO. In addition, the calix[n]arene-functionalized polyamide network may also have great potential for sea water desalination, waste water treatment (e.g., heavy metal and organic pollutants removal), solvent reclamation, and drug purification via pressure-driven process.

## Methods

### Preparation of the cross-linked membrane substrate

The fabrication and cross-linking procedures were described in our previous works^40,41^. Briefly, Matrimid was first dried in a vacuum oven at 70 °C for 24 h before it was dissolved in a mixture of N-methyl-2-pyrrolidinone (NMP) and polyethylene glycol 400 (PEG 400) with a weight ratio of 20:64:16 for Matrimid, NMP, and PEG400. The mixture was stirred at 70 °C for another 24 h to prepare a homogeneous dope and then degassed for at least 1 day prior to casting the membrane substrate on a glass plate, followed by phase inversion in a deionized (DI) water bath. The as-cast substrate was stored in a DI water bath for at least 1 day to complete the phase inversion. To cross-link the substrate, it was cut into a proper size and immersed in an isopropanol (IPA)/water (50/50 wt/wt) solution containing 5 wt% 1,6-hexanediamine (HDA) for 24 h. Afterward, the cross-linked substrate was taken out, washed thoroughly with fresh DI water and stored in DI water for further modifications.

### Fabrication of TFN membranes via interfacial polymerization

Interfacial polymerization between MPD (aqueous phase) and TMC (organic phase) was conducted to form a thin polyamide layer on top of the cross-linked Matrimid membrane substrate, as depicted in Fig. 1c. Briefly, the cross-linked membrane substrate was first immersed into a 2 wt% MPD aqueous solution containing 0.2 wt% SDS for 2 min. For each TFN membrane containing STCAss or SCA, a predetermined amount of calixarene was added into the MPD solution. Afterward, the membrane was taken out and the extra MPD solution on the surface was wiped with filter papers. A hexane solution of 0.1 wt% TMC was then deposited onto the top of the membrane for 1 min, followed by air-drying for 5 min to form the dense polyamide layer. The prepared TFC and TFN membranes were then rinsed with ethanol and immersed in ethanol prior to further testing. The prepared membranes were denoted as TFC-0 (pristine), TFN-STCAss-0.5, TFN-STCAss-1, TFN-STCAss-1.5, TFN-STCAss-2, and TFN-SCA-1.5, where 0.5, 1, 1.5, and 2 refer to the weight percentages of STCAss or SCA in the MPD solutions.

To prepare free-standing polyamide films, an MPD aqueous solution of ~10 ml with the corresponding compositions and a TMC/hexane solution of ~15 ml were prepared. Firstly, the MPD aqueous solution was poured into a petri dish and allowed to stabilize the liquid surface. Subsequently, the TMC/hexane solution was added dropwise on the top surface of the MPD solution. The petri dish was then covered with a lid to prevent the hexane evaporation and stabilize the film growth. After 24 h, the petri dish was drained and the resultant thin film was rinsed several times with ethanol to remove the excess monomers. It was then vacuum dried for further characterizations.

### Solvent reclamation through OSFO

The solvent reclamation was conducted using a lab-scale OSFO unit with solvent resistant tubing and pumps. Both draw and feed solutions were circulated counter-currently using the pumps at volumetric flows of 0.2 L min^−1^. Two operating modes; namely, FO mode (i.e., the selective layer facing the feed solution) and PRO mode (i.e., the selective layer facing the draw solution), were studied. The solvent flux (*J*_w_, LMH) and reverse solute flux (*J*_s_, gMH) were determined using Eqs. (1) and (2), respectively.

1 $$J_{\mathrm{w}} = \frac{{\Delta m}}{{\rho \Delta t}}\frac{1}{{A_{\mathrm{m}}}}$$

2 $$J_{\mathrm{s}} = \frac{{\Delta C_{\mathrm{t}}V}}{{\Delta t}}\frac{1}{{A_{\mathrm{m}}}}$$

Where Δ*m* (g) is the absolute weight loss in the feed side or the absolute weight gain in the draw side, *ρ* (g cm^−3^) is the solvent density, Δ*t* (h) is the testing duration of 2 h, *A*_m_ (cm^2^) is the effective contact area of 4 cm^2^, Δ*C*_t_ (g L^−1^) is the change of solute concentration in the feed solution, and *V* (L) is the volume of the feed solution.

In OSFO tests, LiCl was employed as the draw solute and was dissolved in pure ethanol at a concentration of 2 M. Meanwhile, the pure ethanol was used as the feed solution. The weight gain of the feed solution was monitored using a balance (A&D Company, Ltd, Japan) connected to a data log on a computer. The reverse solute flux was determined using a conductivity meter (Metrohm, Switzerland), where the calibration curve was attained prior to the tests.

To demonstrate the feasibility of concentrating pharmaceuticals and recovering organic solvents using the developed TFN membranes in OSFO processes, tetracycline and paracetamol/ethanol solutions of 2000 ppm were used as the model feed solutions. The FO mode was chosen as it had a lower reverse solute flux and a less fouling tendency than the PRO mode^42^. The membrane rejection to tetracycline/paracetamol, *R*_f_ (%), was defined as follows:

3 $$R_{\mathrm{f}} = \left( {1 - \frac{{C_{\mathrm{d}} \times V_{\mathrm{d}}/V_{\mathrm{p}}}}{{C_{\mathrm{f}}}}} \right) \times 100{\mathrm{\% }}$$

Where *C*_d_ (g L^−1^) is the tetracycline/paracetamol concentration in the draw solution at the end of each OSFO test, *V*_d_ (mL) is the final volume of the draw solution, *V*_p_ (mL) is the volume of the permeate, and *C*_f_ (g L^−1^) is the tetracycline/paracetamol concentration in the feed solution. The tetracycline and paracetamol concentrations were determined by a UV-Vis spectrophotometer (Pharo 300, Merck) according to the Beer–Lambert law.

### Transport properties

The pure solvent permeance (*A*, L m^−2^ h^−1^ bar^−1^, LMH/bar) and salt rejection (*R*_s_, %) were determined by testing the membranes under a transmembrane pressure (Δ*P*) of 10 bar in dead-end cells at room temperature, as described in previous publications^43,44^. The feed solutions were made of 200 ppm LiCl, NaCl and KCl in ethanol. The concentrations of salt in the feed (*C*_f_, g L^−1^) and permeate (*C*_p_, g L^−1^) were determined using a conductivity meter (Metrohm, Switzerland). The pure solvent permeance and solute rejection *R*_s_ were calculated by Eqs. (4) and (5), respectively.

4 $$A = \frac{{\Delta V}}{{\Delta t}}\frac{1}{{A_{\mathrm{m}}\Delta P}}$$

5 $$R_{\mathrm{s}} = \left( {1 - \frac{{C_{\mathrm{p}}}}{{C_{\mathrm{f}}}}} \right) \times 100\%$$

Where Δ*V* (L) is the permeate volume, Δ*t* (h) is the testing duration, *A*_m_ is the effective area of the membrane, and Δ*P* is the applied transmembrane pressure.

Membranes were also tested using water, methanol, and ethanol under a Δ*P* of 10 bar in dead-end cells at room temperature to further understand the solvent transport mechanism inside the pristine and functionalized polyamide layers. The permeability (*P*, cm^2^ s^−1^ bar^−1^) of membranes was calculated using Eq. (6)^45^.

6 $$P = A \times \Delta x$$

Where Δ*x* (m) is the membrane thickness. As the transport resistance of the developed membrane was mainly determined by the polyamide selective layer, the thickness of the polyamide layer was applied here.

The solubility of a membrane was measured using the solvent evaporation method as described previously^9^. Briefly, once the free-standing polyamide thin film was fabricated, it was dried in a vacuum oven to remove the moisture. The film was quickly weighed (*m*_o_) and immersed in an excessive solvent for 1 week to ensure it being fully saturated. After that, the solvent was allowed to evaporate at room temperature and the weight change was recorded as a function of time. Generally, the weight profile would display three weight loss rates, which indicated the solvent evaporation from (1) the membrane surface, (2) inside the membrane, and (3) the cease of evaporation. Therefore, the difference between the turning point (*m*_w_) of the first and second slopes with the final weight (*m*_d_) is considered as the solvent uptake by the membrane. The solubility of the polyamide thin film (*S*, gram solvent per gram membrane, g g^−1^) was calculated using Eq. (7).

7 $$S = \frac{{m_{\mathrm{w}} - m_{\mathrm{d}}}}{{m_{\mathrm{o}}}}$$

Based on D.R. Paul’s paper^46^, the classical solution–diffusion model works for the case where flux is linearly dependent on transmembrane pressures (Supplementary Fig. 5), and thus the diffusivities (*D*, cm^2^ s^−1^) of the selected solvents could be determined as follows:

8 $$D = \frac{{P \cdot RT}}{{C_{\mathrm{s}} \cdot \bar V_{\mathrm{s}}}}$$

Where *C*_s_ (g m^−3^) is the solvent concentration inside the membrane and $\overline {V_{\mathrm{s}}}$ (m^3^ mol^−1^) is the partial molar volume of the respective solvent.

## Supplementary information

Supplementary Information

## Data Availability

The authors declare that all data supporting the findings of this study are available within the paper and its supplementary information files.
