# Supplementary material for: Molecularly tunable thin-film nanocomposite membranes with enhanced molecular sieving for organic solvent forward osmosis
Source: Nat Commun. 2020 Mar 5;11:1198. doi: 10.1038/s41467-020-15070-w (PMC7057969; doi:10.1038/s41467-020-15070-w)
Supplement: Supplementary file 1 — Supplementary Information [file 41467_2020_15070_MOESM1_ESM.pdf]

Supplementary Information

Molecularly tunable thin-film nanocomposite membranes with enhanced molecular sieving for organic solvent forward osmosis

Li et al.

Supplementary Methods

Materials

To fabricate the membrane substrate, a commercially available polyimide, Matrimid® 5218, was purchased from Vantico Inc. N-methyl-2-pyrrolidinone (NMP, Merck) and polyethylene glycol 400 (PEG400, Acros Organics) were chosen as the solvent and pore former, respectively. 1,6-Hexanediamine (HDA, 98%, Sigma Aldrich) was used to cross-link the substrate and improve the chemical stability. M-Phenylenediamine (MPD, >99%, Tokyo Chemical Industry), sodium dodecyl sulfate (SDS, >99%, Sigma-Aldrich), and trimesoyl chloride (TMC, >98%, Sigma-Aldrich) were employed in the interfacial polymerization reaction. 4-Sulfothiacalix[4]arene sodium salt (STCAss, >98%) and 4-sulfocalix[4]arene hydrate (SCA, >94%) were procured from Tokyo Chemical Industry. Lithium chloride (LiCl, >99%, Sigma-Aldrich) and tetracycline (≥ 98%, Sigma-Aldrich) were acquired as the draw solute and model feed solute, respectively. The organic solvents, such as methanol, ethanol and hexane, were provided by VWR Inc.

Membrane characterizations

The surface morphology of the prepared TFC and TFN membranes was characterized by a field emission scanning electron microscope (FESEM, JEOL JSM-6700F). The membrane samples were freeze-dried and fractured in liquid nitrogen, followed by coating with a platinum layer using a JEOL JFC-1300 platinum coater. The thickness of the polyamide layer was determined from FESEM images by using an analytic software. The surface roughness of the polyamide layer was measured by an atomic force microscope (AFM, Bruker Dimension ICON) under the tapping mode. The chemical functionalities of membranes and nano-fillers were elucidated by Fourier Transform Infrared Spectroscopy (FTIR, Bruker) under the attenuated total reflectance (ATR) mode. The surface atomic concentrations were determined using X-ray photoelectron spectroscopy

(XPS, Kratos AXIS UltraDLD) with a monochromatized Al Kα X-ray source (1486.71 eV, 5 mA, 15 kV). In order to minimize the interference from membrane substrates, the free-standing polyamide films were used for both ATR-FTIR and XPS characterizations.

Doppler broadening energy spectroscopy (DBES) is an advanced tool to detect the microstructural variation of each membrane as a function of positron penetration depth [1,2]. A variable mono-energy slow positron beam was generated by a ^{22}Na source in positron annihilation spectroscopy (PAS). A total of 30 DBES spectra were collected at different incident energies between 0-27 KeV. Both S- and R-parameters of DBES were recorded to analyze the evolution of free volume and pore size of the prepared membranes. The mean depth (Z, nm) was correlated to the varied incident energy (E_+, KeV) according to Equation (1):

$$Z(E_+) = \frac{40}{\rho} E_+^{1.6} \tag{1}$$

where ρ (g cm^{-3}) is the density of the polyamide layer, which is taken as 1.3 g cm^{-3} [3].

Supplementary Table 1. Surface compositions of TFC-0, TFN-STCAss-1.5 and TFN-SCA-1.5.

	C 1*s* (At%)	O 1*s* (At%)	N 1*s* (At%)	S 2*p* (At%)	C/N	O/N	S/N
TFC-0	75.72	11.65	12.62	-	6	0.92	-
TFN-STCAss-1.5	74.74	15.71	9.21	0.34	8.11	1.71	0.037
TFN-SCA-1.5	75.76	14.92	9.06	0.26	8.36	1.65	0.029

Supplementary Table 2. Peak area percentages of N 1s for TFC-0, TFN-STCAss1.5 and TFN-SCA-1.5.

	Percentage (%)		
	Primary amine	Secondary amine	Quaternary amine
TFC-0	5.00	84.40	11.60
TFN-STCAss-1.5	8.52	76.96	14.52
TFC-SCA-1.5	7.39	73.96	18.65

Supplementary Table 3. Surface roughnesses of the pristine TFC and TFNs with different STCAss loadings.

Membrane	TFC-0	TFN-STCAss-0.5	TFN-STCAss-1	TFN-STCAss-1.5	TFN-STCAss-2
Rq (nm)	100.00 ± 3.95	75.33±6.19	52.83±2.05	44.80±3.30	86.13±7.71
Ra (nm)	80.33±3.49	60.03±4.33	42.03±1.45	36.27±2.05	65.37±6.50

Supplementary Table 4. Surface compositions of the pristine TFC and TFNs as a function of STCAss loading.

	C 1*s* (At%)	O 1*s* (At%)	N 1*s* (At%)	S 2*p* (At%)	C/N	O/N	S/N
TFC-0	75.72	11.65	12.62	-	6	0.92	-
TFN-STCAss-0.5	80.20	9.83	9.76	0.21	8.21	1.07	0.021
TFN-STCAss-1	77.36	12.96	9.46	0.22	8.18	1.37	0.023
TFN-STCAss-1.5	74.74	15.71	9.21	0.34	8.11	1.71	0.037
TFN-STCAss-2	74.66	14.55	9.70	1.10	7.7	1.5	0.11

Supplementary Table 5. Comparison of OSFO and OSRO for paracetamol and tetracycline.

Mode	Feed solutions	Flux (LMH)	Rejection (%)
OSFO*	Paracetamol/ethanol	3.41	96.1
	Tetracycline/ethanol	3.26	99.6
OSRO^	Paracetamol/ethanol	3.21	79.0
	Tetracycline/ethanol	2.95	97.1

*OSFO: 2M LiCl/ethanol solution as draw solution, 2 g L^{-1} feed solutions;
^OSRO: 10 bar as operating pressure, 0.2 g L^{-1} feed solutions.

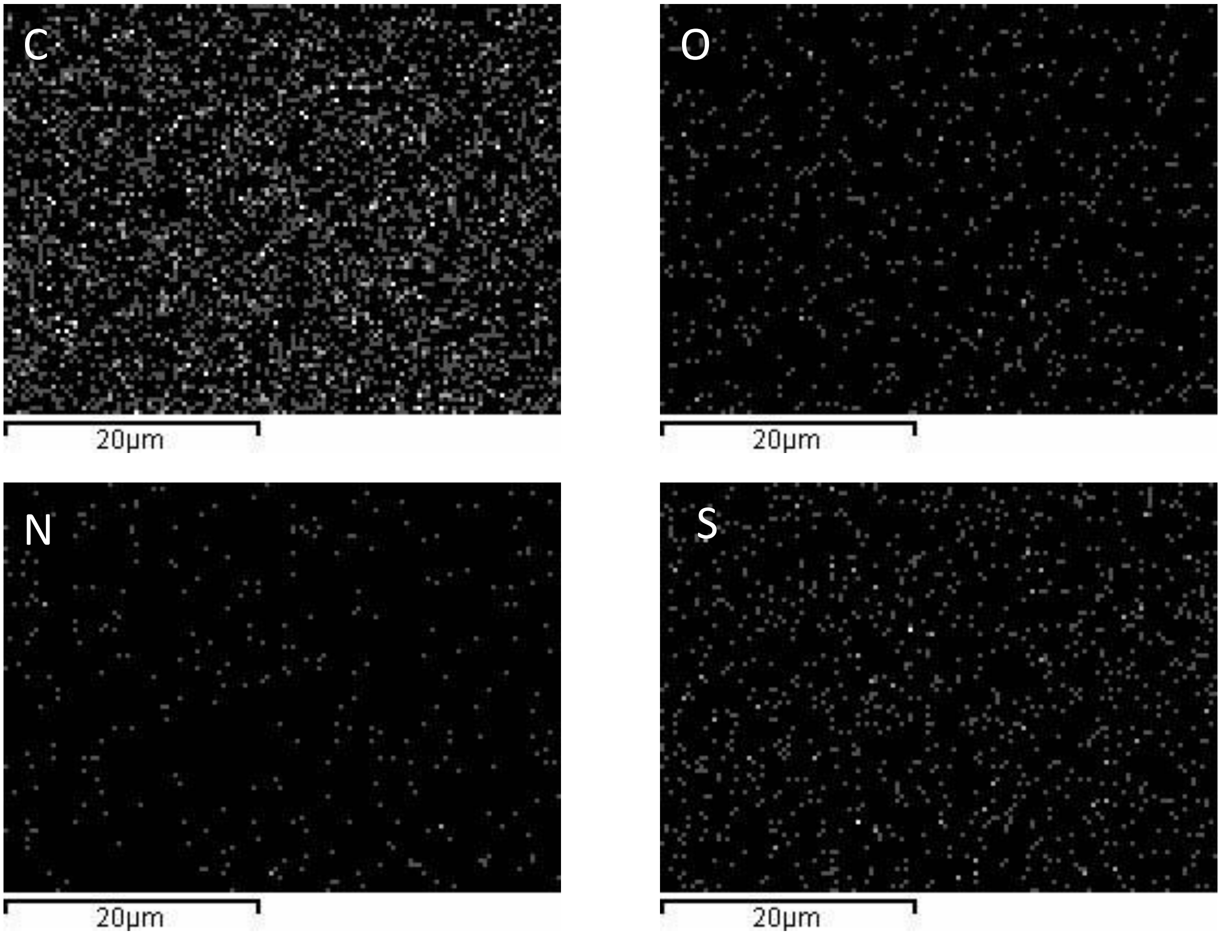

Supplementary Fig. 1. EDX mapping of elements in TFN-STCAss-1.5. The upper-left image is the EDX mapping of C; upper-right image is the mapping of O; lower-left image is the mapping of N and the lower-right image is the mapping of S.

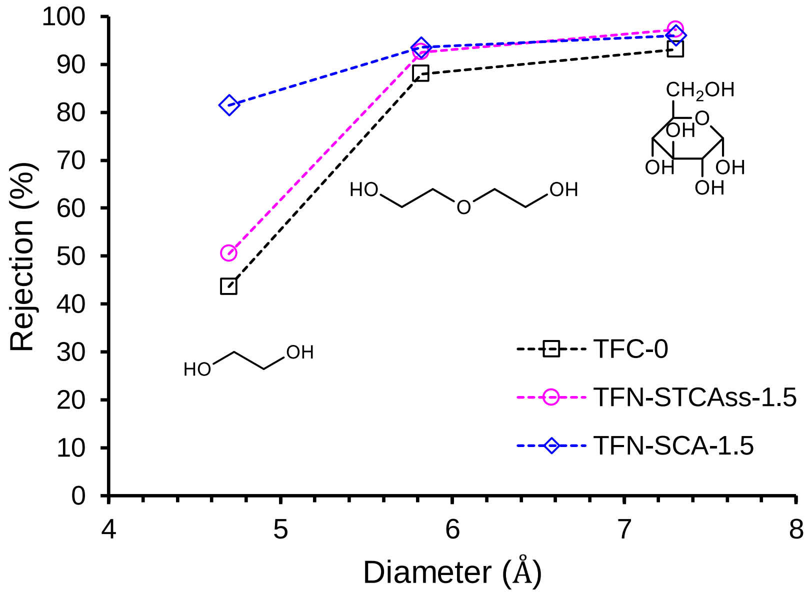

Supplementary Fig. 2. Rejections towards EG, DEG and glucose of the TFC-0, TFN-STCAss-1.5, TFN-SCA-1.5. The diameters of EG, DEG and glucose are 4.7 Å, 5.82 Å and 7 Å, respectively.

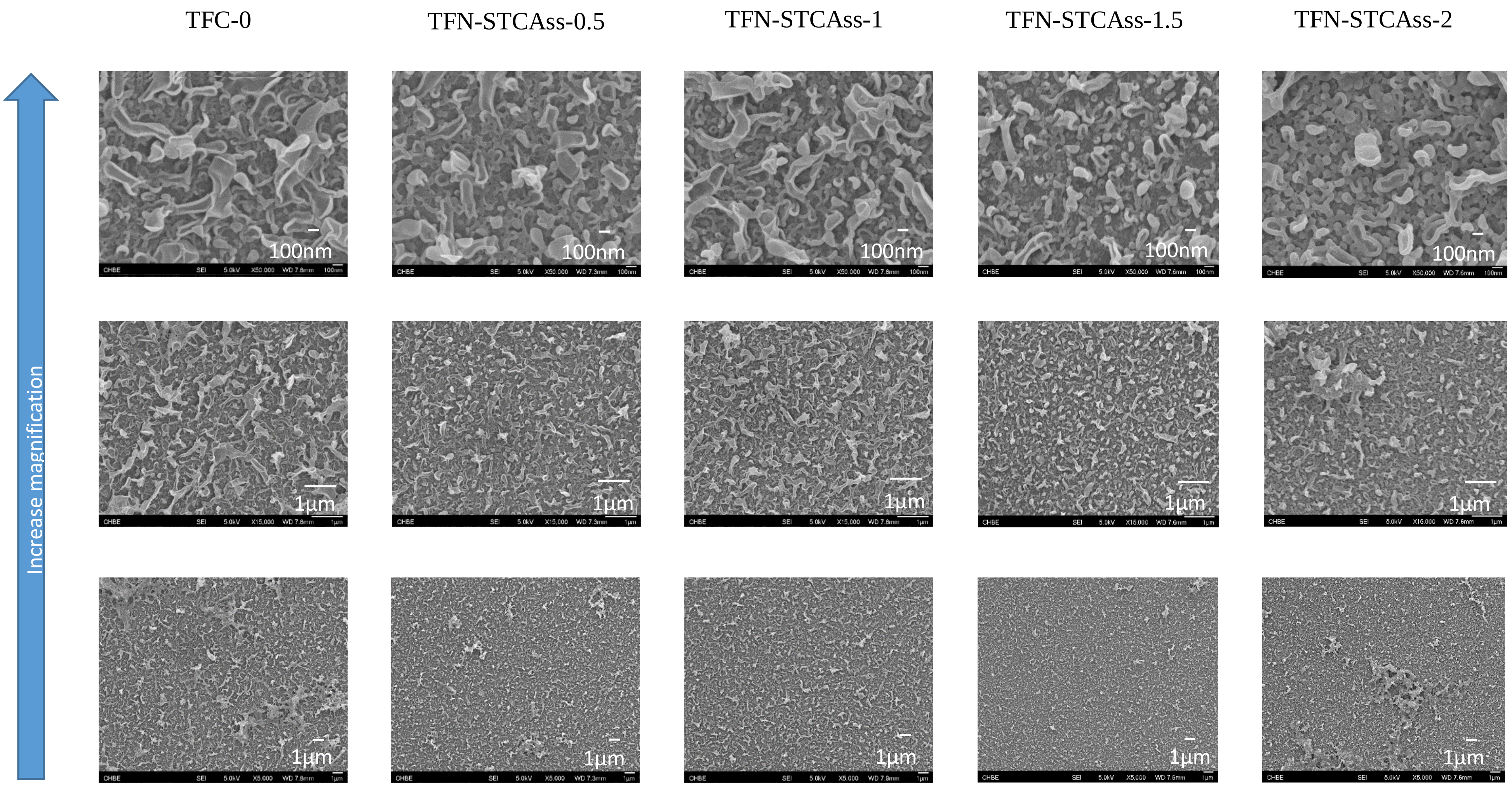

Supplementary Fig. 3. FESEM images of pristine TFC and TFNs with different STCAss loadings. The image magnification is increased from bottom to top.

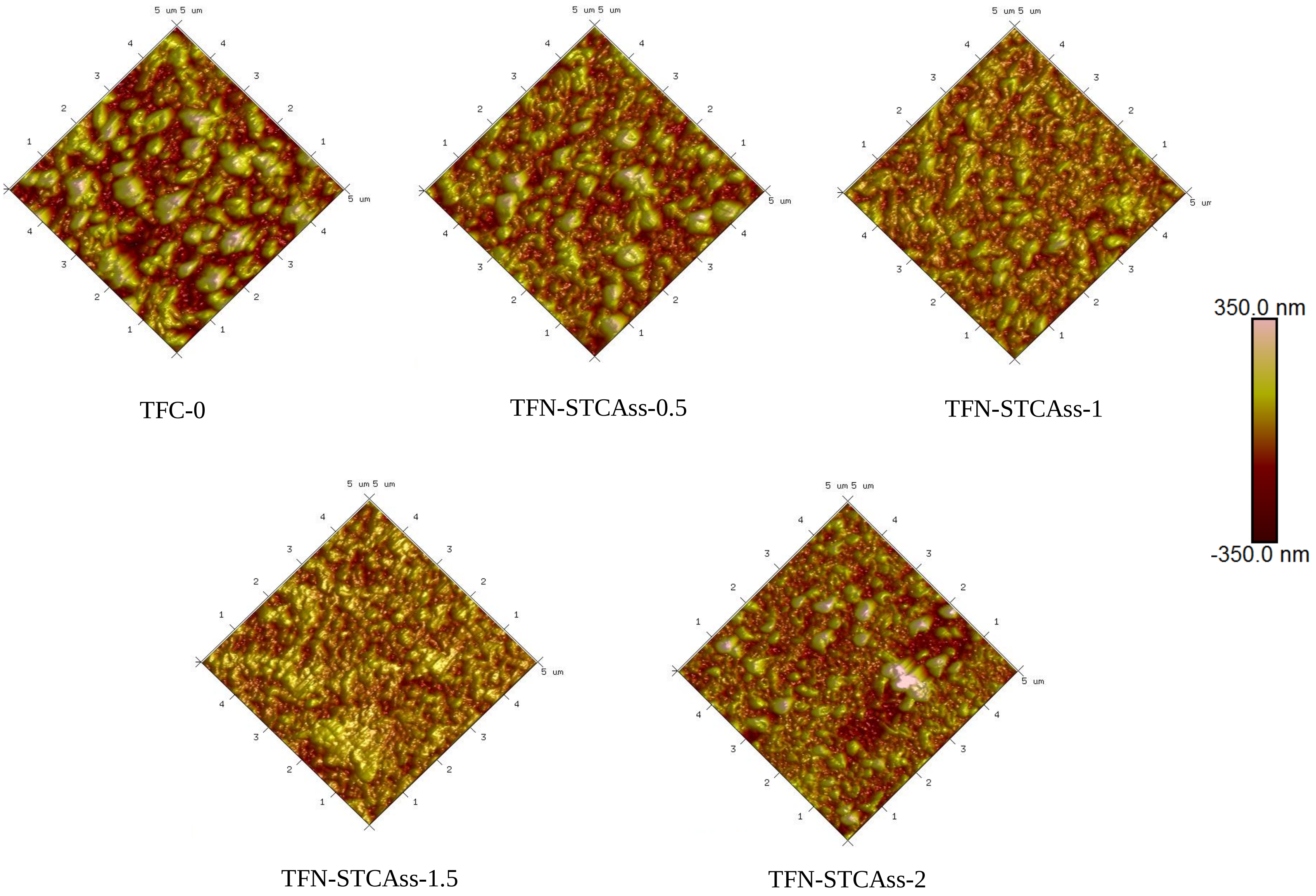

Supplementary Fig. 4. AFM images of pristine TFC and TFN with different STCAss loadings. The right inset is the height profile.

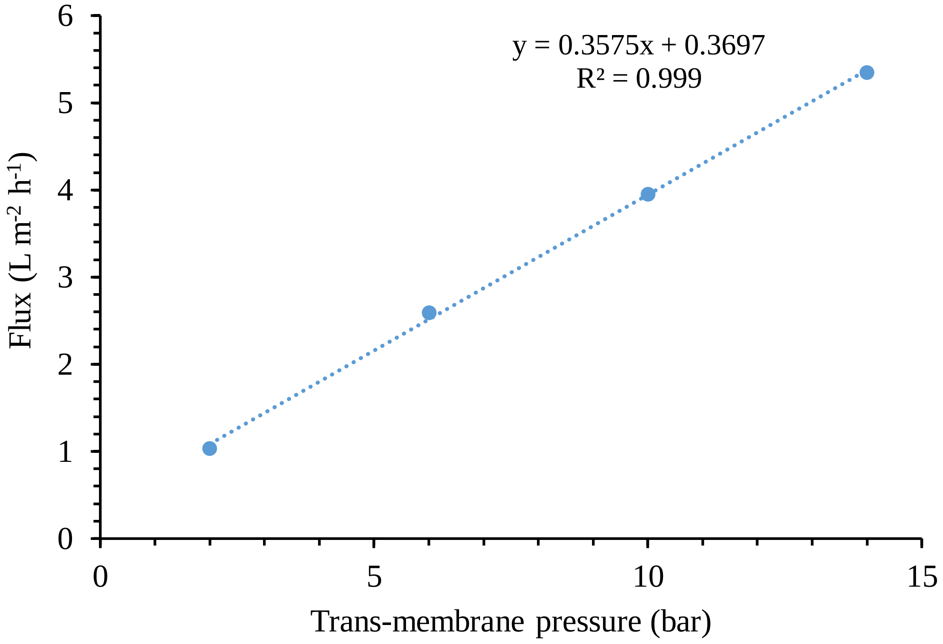

Supplementary Fig. 5. Flux vs. trans-membrane pressure for TFN-STCAss-1.5 in ethanol. The linear correlation equation is inserted above.

Supplementary References

1 Chen, H. *et al.* Free-volume depth profile of polymeric membranes studied by positron annihilation spectroscopy: Layer structure from interfacial polymerization. *Macromolecules* **40**, 7542-7557 (2007).

2 Jean, Y. C. *et al.* Applications of positron annihilation spectroscopy to polymeric membranes. *Desalination* **234**, 89-98 (2008).

3 Ben-David, A., Bason, S., Jopp, J., Oren, Y. & Freger, V. Partitioning of organic solutes between water and polyamide layer of RO and NF membranes: Correlation to rejection. *J. Membr. Sci.* **281**, 480-490 (2006).
